# Clinical Utility of an FDA-Authorized Artificial Intelligence Imaging Platform in Interstitial Lung Disease Diagnosis

**DOI:** 10.3390/diagnostics16152445

**Published:** 2026-08-03

**Authors:** Arjun Prakash Tambe, Ryan D. Boente, Gautam George, Fayez Kheir, Omid Tahamtani Omran, Kavitha C. Selvan

**Affiliations:** 1 Division of Pulmonary and Critical Care Medicine, Department of Medicine, Northwestern University, Chicago, IL 60611, USA; 2 Division of Pulmonary, Critical Care, Allergy and Occupational Medicine, Department of Medicine, Indiana University, Indianapolis, IN 46202, USA; 3 Jane and Leonard Korman Respiratory Institute, Division of Pulmonary, Allergy and Critical Care Medicine, Sidney Kimmel Medical College, Thomas Jefferson University, Philadelphia, PA 19107, USA; 4 Division of Pulmonary and Critical Care Medicine, Department of Medicine, Massachusetts General Hospital, Harvard Medical School, Boston, MA 02114, USA; 5 Independent Researcher, Los Angeles, CA 90095, USA

**Keywords:** interstitial lung disease, idiopathic pulmonary fibrosis, artificial intelligence, chest imaging, computed tomography, CT, antifibrotic, lung biopsy

## Abstract

**Background/Objectives:** The diagnosis of interstitial lung disease (ILD) is challenging and frequently delayed. Clinically accessible and minimally invasive diagnostic tools are needed to expedite the diagnosis of ILD while minimizing risk to patients. Fibresolve is an imaging artificial intelligence (AI) tool recently approved by the Food and Drug Administration (FDA) for use in ILD diagnosis and made available to clinicians. The objective of this study was to describe its utility in clinical practice. **Methods:** We conducted a prospective, observational study of patients across the United States (US) in whom Fibresolve was utilized during routine clinical practice between July 2024 and June 2026. Information on patient demographics, Fibresolve test results (positive = suggestive of idiopathic pulmonary fibrosis (IPF); negative = unsupportive of IPF), and disease management pre- and post-Fibresolve utilization were collected, including the use of ILD-specific pharmacotherapy and planned and/or completed invasive diagnostic procedures. **Results:** There were 209 patients that underwent evaluation with Fibresolve across 47 medical centers. Of those, 16 were academic centers (*n* = 57 patients), and 31 were community-based (*n* = 152 patients). Fibresolve was positive in 89/209 (42.6%) patients. The percentage of patients receiving ILD-specific pharmacotherapy increased significantly following Fibresolve utilization (12.5% to 44.4%, *p* < 0.001). Pre-Fibresolve, 59/154 (38.3%) patients had an invasive diagnostic procedure planned; post-Fibresolve, only 12/77 (15.6%) patients had an invasive procedure performed (*p* < 0.001). **Conclusions:** In this real-world study evaluating the use of an FDA-authorized imaging tool for ILD diagnosis in diverse clinical practices across the US, we found that following utilization of Fibresolve, there was a statistically significant increase in the percentage of patients receiving guideline-directed therapy for ILD, and previously-planned invasive diagnostic procedures were avoided in many cases. These findings support the use of Fibresolve as an adjunct for ILD diagnosis in clinical practice.

## 1. Introduction

The diagnosis of interstitial lung disease (ILD) is challenging, in part due to the overlap in radiologic pattern across diverse subtypes of disease. Within the same radiologic pattern of disease, first-line guideline-recommended treatment can vary significantly. For example, idiopathic pulmonary fibrosis (IPF), connective tissue disease-related ILD (CTD-ILD), and hypersensitivity pneumonitis (HP) may all present with a radiologic usual interstitial pneumonia (UIP) pattern; however, IPF is treated with antifibrotic medications, CTD-ILD is typically treated with immunosuppressive medications, and HP is typically managed with antigen avoidance and/or immunosuppressive therapy [1,2,3,4,5]. Given the heterogeneity in first-line treatment recommendations across ILD subtypes, establishing an accurate diagnosis is critical. Unfortunately, even when the correct diagnosis is made, it is frequently delayed by months to years, and time lost to diagnostic delays can lead to irreversible loss of lung function [6,7,8,9]. This paradigm is best illustrated by IPF, the most common subtype of idiopathic ILD [10]. IPF carries a median survival of only 3–5 years after diagnosis, and left untreated leads to an average loss of approximately 200 mL in forced vital capacity (FVC) annually [11,12,13,14,15,16]. Currently available treatments can slow the progression of disease, but no therapy has been shown to improve lung function in IPF patients [14,17]. Thus, accurate diagnosis and timely treatment initiation are vital to prevent the development and progression of irreversible pulmonary fibrosis [18].

The gold standard for ILD diagnosis is multidisciplinary discussion (MDD) at an expert center, during which clinical history, laboratory data, chest imaging, and histopathology (when available) are reviewed by expert physicians [19]. Unfortunately, MDD is available predominantly at academic centers, and wait times for a consultation may be substantial. As a result, many patients with ILD are initially evaluated and managed in the community by primary care providers and general pulmonologists, who may have less comfort in recognizing and managing these rare diseases [20]. Community-based centers experience lower interobserver agreement between clinicians on imaging patterns, pathology interpretation, and clinical diagnosis of ILD subtype [21]. Additionally, community-based physicians are more likely than academic physicians to assign a final diagnosis of IPF, despite the similarly high prevalence rates of other types of ILD, such as CTD-ILD [21,22]. Further highlighting the diagnostic uncertainty seen outside of academic centers, survey data from patients with ILD showed that 75% of respondents consulted with at least three physicians prior to achieving a final diagnosis, and the majority reported that expert consultation was the most important contributing factor in obtaining a clear diagnosis [6]. Diagnostic uncertainty and delayed access to tertiary care centers has been shown to negatively impact patient outcomes in IPF, including mortality, independent of disease severity [20]. The barriers to timely expert evaluation highlight a need for tools that can support clinicians in making an accurate ILD diagnosis across diverse practice settings.

Over the past few decades, advancements in computed tomography (CT) imaging techniques have led to significant improvement in the non-invasive classification of ILD. High-resolution CT scanning (HRCT) was introduced in the 1980s, offering greater ability to visualize fine detail within the lung [23]. By the early 21st century, multi-detector CT (MDCT) was introduced, enabling the acquisition of thin CT sections covering the whole chest in a single breath hold [24]. Simultaneously, the historic belief that lung biopsy was the reference standard in ILD diagnosis became replaced by a growing understanding that histopathology is not infallible [25]. With these advancements in CT technique and more widespread utilization, CT imaging has become an increasingly important diagnostic test in the workup of ILD. The identification of certain morphologic patterns on chest CT can now significantly narrow the differential diagnosis of specific ILD subtypes and obviate the need for invasive tissue sampling [26,27,28].

Despite the substantial advances in imaging techniques, up to 61% of patients with ILD are still referred for invasive procedures—such as bronchoalveolar lavage, transbronchial biopsy, or surgical lung biopsy—to improve diagnostic confidence [6,29]. Though invasive procedures may allow for a final pathobiological diagnosis, it is important to recognize that they also introduce morbidity and mortality. Risks of transbronchial biopsy include airway bleeding and pneumothorax. Additionally, there is a significant risk of obtaining a non-diagnostic specimen: the diagnostic yield for cryobiopsy and traditional forceps biopsy in the workup of diffuse lung disease was recently reported to be 72% and 62.5%, respectively [30]. Surgical lung biopsy is considered even higher-risk than using a bronchoscopic approach, with 30-day post-operative mortality of at least 2–3% [31,32,33,34]. When comparing utilization of invasive procedures for ILD diagnosis based on practice setting, general pulmonologists were found to use bronchoalveolar lavage, transbronchial biopsy, and surgical lung biopsy significantly more frequently than ILD specialists, suggesting lower diagnostic confidence in imaging interpretation [29]. This is supported by the lower inter-observer agreement on radiologic patterns seen amongst non-ILD specialists when compared to specialists [35]. However, inter-reader disagreement in CT interpretation is common even among experienced thoracic radiologists, occurring in approximately half of cases [36]. These challenges have prompted the development of artificial intelligence (AI) platforms to aid non-invasive ILD diagnosis.

Fibresolve (Version 1, IMVARIA, Inc., Berkeley, CA, USA) is a novel, recently FDA-authorized AI software for the diagnosis of ILD. Specifically, Fibresolve was designed to provide a diagnostic subtype classification in suspected cases of ILD and serve as an adjunct in the diagnosis of IPF. As previously described, the Fibresolve platform analyzes chest CT images using a deep learning convolutional neural network (CNN) algorithm to non-invasively detect findings consistent with IPF. Importantly, unlike other IPF classifier tools, Fibresolve analysis is not limited to the pre-defined features of UIP [37,38,39,40,41]. Fibresolve produces a binary text output: positive (suggestive of IPF) or negative (unsupportive of IPF). The software was initially trained using a multi-center dataset of over 2000 cases of ILD; it subsequently underwent algorithm tuning using a separate dataset of 295 patients across multiple sites in the United States (US) [37]. Data obtained for training and tuning included clinical history, CT images, lung biopsy results, and final MDD diagnosis. The percentage of patients with an MDD consensus diagnosis of IPF was 44% and 25% in the initial training dataset and subsequent tuning dataset, respectively. In the tuning dataset, 70% of patients had lung biopsy results, in addition to clinical history, CT images, and MDD diagnosis. Initial validation data using a separate registry demonstrated that Fibresolve had a sensitivity of 67% and specificity of 90% for the diagnosis of IPF, using MDD consensus as the diagnostic gold standard [37]. In a follow-up validation study of Fibresolve applied retrospectively to 300 patients with ILD, the platform achieved a sensitivity of 41% and specificity of 87% for the diagnosis of IPF, again using MDD as the diagnostic gold standard, with most patients also having lung biopsy results [42]. Subsequently, Fibresolve was validated for the diagnosis of IPF in cases that lacked a definite UIP pattern on chest CT, achieving a sensitivity of 76.5%. Among a subset of patients who had undergone lung biopsy for diagnostic confirmation, the sensitivity of Fibresolve was similar (74.5%) [43]. Finally, in a fourth validation study of Fibresolve retrospectively performed on 295 patients with a MDD consensus diagnosis of ILD, Fibresolve achieved a sensitivity of 67% and specificity of 90% for IPF diagnosis. In a subgroup of 236 patients who lacked a definite or probable UIP pattern on CT imaging, Fibresolve performed similarly, with a sensitivity of 65% and specificity of 94% relative to MDD reference standard, with 73% of patients having lung biopsy results available during MDD [44]. Overall, these four validation studies demonstrated that Fibresolve has moderate sensitivity but consistently high specificity (86–90+%) for IPF diagnosis, even when applied to cases lacking a UIP imaging pattern [37,42,43,44]. Beyond its ability to identify clinical cases of IPF, Fibresolve has also been shown to predict mortality in patients with ILD across diverse subtypes of disease and offer increased cost effectiveness relative to utilizing lung biopsies for IPF diagnosis [45,46].

Together, existing data suggest that Fibresolve may be a useful adjunct in the diagnostic evaluation of ILD, particularly for patients in whom a diagnosis of IPF is suspected, but diagnostic confidence is lacking. A positive result may expedite the diagnosis of IPF and obviate the need for invasive procedures, while a negative result may heighten suspicion for alternative diagnoses and ultimately impact clinical management. The purpose of this study was to evaluate the utility of Fibresolve in active clinical use across diverse clinical centers in the US.

## 2. Materials and Methods

We conducted a prospective, observational study evaluating real-world clinical use of Fibresolve across academic and community centers in the US between July 2024 and June 2026. Fibresolve (Version 1) was utilized as an adjunct to ILD diagnosis at the discretion of the evaluating physician, consistent with the intended use of its FDA authorization. All patients were being evaluated for ILD, but the specific subtype was not yet established. Patients were included in the study if their physician used Fibresolve during routine diagnostic workup. There were no specific exclusion criteria. Data were collected during ongoing clinical use and de-identified prior to analysis. Pre-Fibresolve data was obtained at the time the Fibresolve test was ordered. The timeframe for collecting follow-up data (relative to Fibresolve testing date) was variable, depending on the scheduling of subsequent medical appointments. This study was reviewed and deemed exempt by Argus Independent Review Board.

Demographic data, including age, sex, smoking status, and percent predicted FVC, were collected prospectively at the time of Fibresolve test ordering via a standardized intake form completed by the ordering physician. Required pre-testing information included patient age, patient sex, and practice setting. Optional information completed at the discretion of the referring clinician included: percent predicted FVC, smoking status, and management plan prior to Fibresolve utilization, such as prescription of antifibrotic medications and whether diagnostic procedures and/or expert center referral were planned. Following Fibresolve utilization, Fibresolve test result was collected (positive = suggestive of IPF, or negative = unsupportive of IPF) for all participating patients. When available, additional clinical information on management decisions was obtained from the electronic medical record, including treatment initiation and use of invasive diagnostic procedures, such as bronchoscopy (with or without biopsy) and surgical lung biopsy.

Descriptive statistics, including median, percentages, and interquartile range, were used to analyze baseline characteristics of the patient population. The proportion of patients on pharmacotherapy for ILD (including anti-fibrotic medications or immunosuppression) pre- and post-Fibresolve testing was reported; 95% confidence intervals (95% CI) were generated using the Wilson score. A generalized estimating equation (GEE) was used to assess for a significant difference in pharmacotherapy utilization before and after Fibresolve testing. Similarly, the proportion of patients who had an invasive diagnostic procedure planned prior to Fibresolve was compared to the proportion of patients who had an invasive diagnostic procedure completed following Fibresolve utilization, using the Wilson score to generate 95% confidence intervals and a GEE to assess for statistical significance. GEE was chosen because not all patients had data available pre- and post-Fibresolve testing, resulting in different sample sizes but still significant patient overlap between the two groups. To assess for the possibility of selection bias within the subsets of patients with available clinical data, baseline characteristics were compared between patients with data available pre- vs. post-Fibresolve testing (Table A2 and Table A3).

The utilization of ILD-specific pharmacotherapy and invasive diagnostic procedures planned/completed pre- and post-Fibresolve testing was compared, stratified by study center type (academic vs. community-based); 95% confidence intervals were generated using the Wilson score. Among the subgroup of patients with paired pre- and post-Fibresolve data on ILD pharmacotherapy, the proportion of patients on therapy was compared using McNemar’s exact test to assess for a statistically significant difference in utilization following Fibresolve testing, when stratified by Fibresolve test result. Similarly, the proportion of patients on ILD pharmacotherapy was compared using McNemar’s exact test within the subsets of patients at academic and community-based sites, respectively. Among the patients who had a definitive plan for an invasive diagnostic procedure prior to Fibresolve testing and available follow-up data on ultimate management following Fibresolve testing, the proportion of patients who had the invasive procedure deferred was compared when stratified by Fibresolve test result and study center type (academic or community-based). Fisher’s exact test was performed to assess for whether Fibresolve test result led to a statistically significant difference in the rate of procedure deferral.

To assess which factors contributed to the initiation of ILD-specific pharmacotherapy following Fibresolve testing, a multivariable regression analysis was performed. To account for missing FVC and smoking status values and preserve the full analytic sample (*n* = 72), multiple imputation by chained equations (50 imputations) was performed, imputing missing covariates via regression models conditioned on all other covariates and the outcome. A logistic regression model of therapy initiation on age, sex, smoking status, percent predicted FVC, and Fibresolve result was fit across the imputed datasets, pooling estimates using Rubin’s rules. A similar multivariable regression analysis assessing the utilization of invasive diagnostic procedures was not performed due to insufficient sample size for meaningful analysis. For all statistical methods used, a *p*-value less than 0.05 was considered statistically significant.

## 3. Results

### 3.1. Patient and Study Center Characteristics

**Figure 1:**
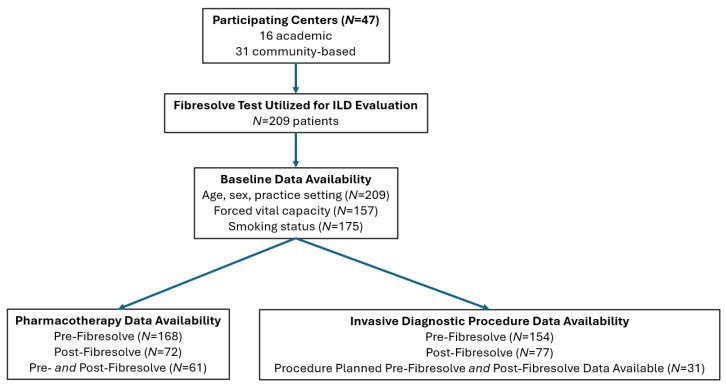
Patient flow diagram. A total of 209 patients underwent Fibresolve utilization at 47 centers across the United States. No patients were excluded, but not all data were available for each patient. Utilization of ILD-specific pharmacotherapy and invasive diagnostic procedures was compared pre- and post-Fibresolve testing. ILD: interstitial lung disease; FVC: forced vital capacity.

In the first two years of clinical use, 209 patients underwent evaluation with Fibresolve across 47 centers in the US (Figure 1). There were 16 academic centers (*n* = 57 patients) and 31 community centers (*n* = 152 patients) represented (Table A1). Patient age, sex, and practice setting were available for all patients (Table 1). Percent predicted FVC, smoking history, and management plan prior to Fibresolve utilization were available for most patients. The median age was 73 years, 83 patients (39.7%) were female, and the median percent predicted FVC was 84%. Most patients were either former smokers (*n* = 95/175, 54.3%) or non-smokers (*n* = 72/175, 41.1%).

### 3.2. Pre-Fibresolve Clinical Management

Prior to Fibresolve utilization, the majority of patients (*n* = 157, 75.1%) had additional imaging studies planned (Table 2). Of 168 patients with available data on pharmacotherapy prescribed prior to Fibresolve utilization, 21 (12.5%) were on antifibrotic therapy. Of 154 patients with available data, 59 (38.3%) had an invasive procedure planned prior to Fibresolve utilization, and 36 (23.4%) were being considered for expert center referral.

### 3.3. Fibresolve Test Results and Comparison of Patient Characteristics Based on Fibresolve Result

Eighty-nine patients (42.6%) had a positive Fibresolve result, suggestive of an IPF diagnosis (Table 1). Patients with a positive result were predominantly male and current or former smokers, compared to a lower frequency of these characteristics in patients with a negative Fibresolve test result. Other baseline characteristics were similar across both groups. The median percent predicted FVC was not different in patients with a positive vs. negative test result (83% [IQR 72–94%] vs. 84% [IQR 71–96%], respectively). The proportion of patients with a reduced FVC (defined as percent predicted FVC ≤ 80%) was also similar across groups (42.3% vs. 42.2%, respectively).

### 3.4. Post-Fibresolve Clinical Management

Following Fibresolve utilization, approximately one-third of patients had available data on clinical management (Table 3). Of 72 patients with available data, 32 (44.4%) were prescribed pharmacotherapy for ILD following Fibresolve utilization. Of 77 patients with available data, only 12 (15.6%) had an invasive diagnostic procedure performed after Fibresolve testing. Although many patients lacked follow-up data, the characteristics of patients with available follow-up data were representative of the characteristics of the entire cohort (Table A2 and Table A3). There were statistically significant differences in the utilization of ILD-specific pharmacotherapy (odds ratio (OR) = 5.84, 95% CI: 3.21–10.60, *p* < 0.001) and invasive diagnostic procedures (OR = 0.26, 95% CI: 0.13–0.52, *p* < 0.001) pre- vs. post-Fibresolve testing. There was not a meaningful difference in the utilization of ILD pharmacotherapy when comparing academic and community-based centers; at both center types, the proportion of patients on pharmacotherapy increased after Fibresolve testing (Table 4). Patients at academic centers had a higher frequency of both invasive diagnostic procedures planned pre-Fibresolve and invasive procedures ultimately completed post-Fibresolve compared to patients at community-based centers (Table 5). Of 51 patients with available data, only three patients (5.9%) at community-based centers underwent an invasive diagnostic procedure following Fibresolve testing.

Sixty-one patients had data available on ILD-specific pharmacotherapy both pre- and post-Fibresolve (Table 6). Of those, 25 (41.0%) had a positive Fibresolve result, similar to the percentage seen in the entire cohort (42.6%). The number of patients on pharmacotherapy for ILD significantly increased from six (9.8%) to 28 (46.7%) following Fibresolve use (*p* < 0.001). Pharmacotherapy utilization was higher among patients with a positive test result (*n* = 16/25, 64.0%) compared to those who had a negative test result (*n* = 12/36, 33.3%). In both groups, the absolute percentage of individuals on pharmacotherapy for ILD increased significantly following Fibresolve testing (*p* < 0.001 in Fibresolve-positive patients; *p* = 0.039 in Fibresolve-negative patients). At academic centers, all six patients with a positive Fibresolve test result initiated ILD pharmacotherapy following Fibresolve utilization. Approximately half (10/19, 52.6%) of patients with a positive Fibresolve test result at community-based centers were on ILD pharmacotherapy following Fibresolve utilization.

Of 59 patients with a documented invasive procedure planned prior to Fibresolve utilization, 31 (52.5%) also had data available on management following Fibresolve testing (Table 7). Sixteen of these (51.6%) had a positive Fibresolve result. All 31 patients had plans to undergo an invasive procedure pre-Fibresolve utilization, but only one-third (*n* = 10/31, 32.3%) ultimately had a procedure performed. Patients with a positive Fibresolve result had a higher frequency of deferring invasive procedures: 81.3% (*n* = 13/16) vs. 53.3% (*n* = 8/15) in patients with a negative Fibresolve result; however, this difference was not statistically significant (*p* = 0.135). At community-based centers, most patients (*n* = 15/17, 88.2%) had invasive procedures deferred following Fibresolve testing, whereas only 42.9% (*n* = 6/14) of patients at academic centers had invasive procedures deferred.

Multivariable regression analysis demonstrated that Fibresolve test result was a statistically significant factor (OR 5.99, *p* = 0.003) correlating with the initiation of pharmacotherapy following Fibresolve testing (Table A4). No significant contribution was observed for age, sex, smoking status, or percent predicted FVC. A similar regression analysis for procedure utilization was deferred due to inadequate sample size.

## 4. Discussion

In this study of over 200 patients undergoing evaluation for ILD across diverse clinical practices in the US, we describe patterns in clinical management following use of an FDA-authorized imaging AI platform to aid in the non-invasive diagnosis of ILD subtype. Our findings show that following utilization of Fibresolve, more patients received guideline-directed treatment for ILD, and many avoided previously-planned invasive diagnostic procedures, such as lung biopsy. These findings were more prominent amongst those with a positive test result, consistent with the platform’s high specificity parameter for IPF diagnosis. Our findings suggest that routine clinical use of this platform has the potential to improve patient outcomes through increased diagnostic confidence in ILD subtype and earlier initiation of guideline-directed therapies, particularly in patients with IPF. In the setting of ongoing rapid expansion in the development and investigation of AI tools for ILD classification, this is the first study to our knowledge reporting how clinical utilization may impact practice patterns in ILD management [47].

In January 2024, Fibresolve received the first FDA authorization of an AI software for diagnostic use in ILD, and since that time, clinical utilization has rapidly increased [48]. More recently, the FDA authorized two additional AI platforms for use in ILD: e-Lung and IQ-UIP [49,50,51,52]. Table A5 summarizes all three platforms. The e-Lung platform provides quantitative support in the examination of CT findings and can identify and quantify lung volume reduction on imaging in patients with known ILD, independent of ILD subtype [49,50,53]. The IQ-UIP platform identifies a radiologic UIP pattern and is indicated for use in passively notifying specialists associated with ILD centers of chest CT scan findings suggestive of a radiologic UIP pattern [41,51,52]. Although both platforms were designed for use in ILD, their specific indications differ from that of Fibresolve. e-Lung and IQ-UIP utilize artificial intelligence to provide clinicians with additional data on CT imaging findings, but they do not directly provide a clinical diagnosis. For example, a UIP pattern identified by the IQ-UIP software could be due to IPF, CTD-ILD, fibrotic hypersensitivity pneumonitis, or drug-induced pneumotoxicity [54]. Rather than identifying a radiologic UIP pattern alone, Fibresolve was trained and validated to support a clinical diagnosis of IPF. Furthermore, while studies have demonstrated the utility of e-Lung and IQ-UIP in controlled research settings, data on their impact on management in clinical practice are lacking. Our study shows that outside of the research setting, utilization of Fibresolve has real-world implications on clinical practice patterns.

Many patients in this study had missing clinical data; however, the patients included in analysis had baseline characteristics that reflected the overall cohort, suggesting that data were missing at random and less prone to selection bias. Patients with a positive Fibresolve result were more frequently men and current or former smokers compared to Fibresolve-negative patients, in concordance with demographic risk factors associated with IPF [55]. The remaining reported clinical characteristics were similar between patients with a positive vs. negative Fibresolve test result. Practice patterns regarding ILD-specific pharmacotherapy were similar between academic and community-based centers. Interestingly, following Fibresolve testing, only three patients (5.9% of patients with available data) at community-based centers underwent an invasive diagnostic procedure; conversely, approximately one-third of patients at academic centers had an invasive diagnostic procedure performed after Fibresolve testing. This trend may be explained by a higher case complexity at academic centers, higher pre-Fibresolve planned procedure rate at academic centers in this study, and/or increased perceived confidence in the Fibresolve result at community-based centers. Notably, while a positive Fibresolve result has previously been validated to be highly specific for IPF, a negative Fibresolve result does not support a specific non-IPF subtype of ILD. Following a negative test result, we suspect clinicians pursued invasive diagnostic testing in certain cases to help determine a final ILD subtype. We also observed a very small number of patients who underwent an invasive diagnostic procedure after a positive Fibresolve result; this may have occurred for additional diagnostic support given the relatively recent FDA approval of Fibresolve, despite available data on test specificity.

The majority of centers included in this study were community-based practices that lack routine access to ILD specialists and expert MDD. A positive Fibresolve test result was followed by avoidance of invasive procedures and initiation of pharmacotherapy for ILD in most patients, suggesting that use improves non-invasive diagnostic confidence and may expedite treatment initiation in IPF. The median percent predicted FVC was not different in patients with a positive vs. negative Fibresolve test result, illustrating that positive results were not simply limited to patients with more advanced fibrotic disease. Additionally, the relatively preserved FVC of patients in this study suggests that Fibresolve has diagnostic utility even in early disease, during which prompt treatment initiation has the greatest potential to impact patient outcomes. Following FDA approval, Fibresolve is readily accessible to clinicians across diverse practice settings, and previous studies have demonstrated that it performs consistently across a variety of CT scanner manufacturers and slice thicknesses [37,42,43,44]. The Fibresolve platform provides binary and easily interpretable results within days, in contrast to often-lengthy wait times for expert center referral and/or procedure coordination [6]. It may be particularly useful at community-based centers without direct access to MDD and/or in early disease, when traditional CT interpretation may be nonspecific.

This early clinical utility study was limited by missing data, specifically related to pre- and post-test medical therapies, procedural planning, and final ILD subtype designation. A minority of patients were missing baseline demographic data, primarily attributable to portions of the Fibresolve test order form that are optional to complete, designed to mitigate administrative burden of platform utilization. Access to data on clinical management following Fibresolve utilization was limited due to the number of study sites, lack of an integrated electronic medical record, and variability in physician documentation. The significant amount of missing post-Fibresolve data may influence the reliability of reported results. As use of Fibresolve continues to expand and additional follow-up data are accrued, the utility of this platform in expediting ILD diagnosis and management will be better characterized. Additional limitations to this study include the possibility of selection bias, as utilization of Fibresolve was based on physician discretion, rather than routinely utilized for all patients presenting for ILD evaluation at each study center. Residual confounding is possible, as metrics not observed in this study may also account for differences in management before and after Fibresolve utilization. Observational bias may have occurred when clinicians made management decisions following Fibresolve testing, without necessarily waiting for a consensus diagnosis from MDD. A positive Fibresolve test result, while suggestive of IPF, may have led to increased utilization of antifibrotic medications in patients with early or mild disease, in whom a watchful waiting approach may have otherwise been employed. Lastly, like other diagnostic tests, Fibresolve may return false-positive or false-negative results. A false-positive result may lead to erroneous initiation of antifibrotic therapy if the correct ILD diagnosis is not subsequently made by the evaluating physician or via MDD. However, the high specificity of Fibresolve (86–90% in previous validation studies) helps to mitigate this risk [37,42,44].

Though this study describes trends in clinical practice patterns before and after Fibresolve utilization, the observational study design limits inference of causality. Future studies could include a time-to-diagnosis metric to quantify how Fibresolve may expedite ILD diagnosis. Expanding to centers outside the United States would be important to validate findings across diverse patient populations and practice settings. Ultimately, longitudinal data on pulmonary function tests, hospitalization rates, and survival compared between patients with and without Fibresolve testing would confirm whether Fibresolve use leads to improvement in long-term clinical outcomes for patients.

## 5. Conclusions

Utilization of the Fibresolve platform across diverse clinical settings in the US was followed by an increase in the proportion of patients on guideline-directed pharmacotherapy for ILD and a decrease in invasive diagnostic testing. Widespread clinical use of Fibresolve may expedite accurate diagnosis and treatment initiation while simultaneously minimizing unnecessary invasive procedures for patients with ILD.

## Figures and Tables

**Table 1:** Baseline patient characteristics and practice setting stratified by Fibresolve test result.

Characteristic	Fibresolve (+)	Fibresolve (−)	Total
Age, median years (IQR) *	74 (69–80)	72 (66–77)	73 (67–79)
Sex, *n* (%) *			
Male	62 (69.7%)	64 (53.3%)	126 (60.3%)
Female	27 (30.3%)	56 (46.6%)	83 (39.7%)
Practice Setting, *n* (%) *			
Academic Center	23 (25.8%)	34 (28.3%)	57 (27.3%)
Community-Based Center	66 (74.2%)	86 (71.7%)	152 (72.7%)
Percent Predicted FVC, median (IQR) †	83% (72–94%)	84% (71–96%)	84% (71–95%)
Smoking Status, *n* (%) ‡			
Current Smoker	2 (2.6%)	6 (6.1%)	8 (4.6%)
Former Smoker	53 (69.7%)	42 (42.4%)	95 (54.3%)
Non-Smoker	21 (27.6%)	51 (51.5%)	72 (41.1%)

* *N* = 209 [89 Fibresolve (+), 120 Fibresolve (−)]; † *N* = 157 [70 Fibresolve (+), 87 Fibresolve (−)]; ‡ *N* = 175 [76 Fibresolve (+), 99 Fibresolve (−)]; Fibresolve (+): patients with a positive Fibresolve result; Fibresolve (−): patients with a negative Fibresolve result; IQR: interquartile range; FVC: forced vital capacity.

**Table 2:** Pre-Fibresolve clinical management plan.

Clinical Management Plan	*n* (%)
Additional imaging planned *	157 (75.1%)
Prescribed antifibrotic therapy †	21 (12.5%)
Invasive procedure planned ‡	59 (38.3%)
Expert center referral planned ‡	36 (23.4%)

* *N* = 209; † *N* = 168; ‡ *N* = 154.

**Table 3:** Pharmacotherapy use and procedural planning/completion pre- and post-Fibresolve utilization.

	Pre-Fibresolve	Post-Fibresolve	*p*-Value
On ILD Pharmacotherapy, *n*/*N* (%) [95% CI]	21/168 (12.5%) [8.3–18.4%]	32/72 (44.4%) [33.5–55.9%]	<0.001
Invasive Procedure Planned/Completed, *n*/*N* (%) [95% CI]	59/154 (38.3%) [31.0–46.2%]	12/77 (15.6%) [9.2–25.3%]	<0.001

The 95% confidence intervals were generated using the Wilson score. *p*-values were calculated using generalized estimating equations (GEE). ILD: interstitial lung disease; CI: confidence interval.

**Table 4:** Pharmacotherapy use pre- and post-Fibresolve utilization stratified by center type.

	On ILD Pharmacotherapy, *n*/*N* (%) [95% CI]		*p*-Value
	Pre-Fibresolve	Post-Fibresolve	
Academic Centers	6/52 (11.5%) [5.4–23.0%]	13/25 (52.0%) [33.5–70.0%]	<0.001
Community-Based Centers	15/116 (12.9%) [8.0–20.2%]	19/47 (40.4%) [27.6–54.7%]	<0.001
Total	21/168 (12.5%) [8.3–18.4%]	32/72 (44.4%) [33.5–55.9%]	<0.001

The 95% confidence intervals were generated using the Wilson score. *p*-values were calculated using generalized estimating equations (GEE).

**Table 5:** Procedural planning/completion pre- and post-Fibresolve utilization stratified by center type.

	Invasive Procedure Planned/Completed, *n*/*N* (%) [95% CI]		*p*-Value
	Pre-Fibresolve	Post-Fibresolve	
Academic Centers	26/47 (55.3%) [41.3–68.6%]	9/26 (34.6%) [19.4–53.8%]	0.032
Community-Based Centers	33/107 (30.8%) [22.9–40.1%]	3/51 (5.9%) [2.0–15.9%]	0.003
Total	59/154 (38.3%) [31.0–46.2%]	12/77 (15.6%) [9.2–25.3%]	<0.001

The 95% confidence intervals were generated using the Wilson score. *p*-values were calculated using generalized estimating equations (GEE).

**Table 6:** Proportion of patients on pharmacotherapy for ILD, stratified by Fibresolve result and study center type.

	**Fibresolve Result**		
**At Academic and Community-Based Centers**	Positive	Negative	Total
On ILD Pharmacotherapy Pre-Fibresolve, *n*/*N* * (%)	1/25 (4.0%)	5/36 (13.9%)	6/61 (9.8%)
On ILD Pharmacotherapy Post-Fibresolve, *n*/*N* * (%)	16/25 (64.0%)	12/36 (33.3%)	28/61 (46.7%)
*p*-value	<0.001	0.039	<0.001
**At Academic Centers Only**			
On ILD Pharmacotherapy Pre-Fibresolve, *n*/*N* † (%)	1/6 (16.7%)	2/16 (12.5%)	3/22 (13.6%)
On ILD Pharmacotherapy Post-Fibresolve, *n*/*N* † (%)	6/6 (100.0%)	5/16 (31.3%)	11/22 (50.0%)
*p*-value	0.063	0.250	0.008
**At Community-Based Centers Only**			
On ILD Pharmacotherapy Pre-Fibresolve, *n*/*N* ‡ (%)	0/19 (0.0%)	3/20 (15.0%)	3/39 (7.7%)
On ILD Pharmacotherapy Post-Fibresolve, *n*/*N* ‡ (%)	10/19 (52.6%)	7/20 (35.0%)	17/39 (43.6%)
*p*-value	0.002	0.219	<0.001

*p*-values were calculated using McNemar’s exact test. * *N* = 61 patients across all centers with paired pre- and post-Fibresolve pharmacotherapy data available. † *N* = 22 patients at academic centers with paired pre- and post-Fibresolve pharmacotherapy data available. ‡ *N* = 39 patients at community-based centers with paired pre- and post-Fibresolve pharmacotherapy data available. ILD: interstitial lung disease.

**Table 7:** Proportion of patients that deferred invasive diagnostic procedures following Fibresolve utilization, stratified by Fibresolve test result and study center type.

Invasive Procedure Deferred Post-Fibresolve *	Fibresolve Result		
	Positive	Negative	Total
Patients at Academic Centers, *n*/*N* * (%)	3/5 (60.0%)	3/9 (33.3%)	6/14 (42.9%)
Patients at Community-Based Centers, *n*/*N* * (%)	10/11 (90.9%)	5/6 (83.3%)	15/17 (88.2%)
Total, *n*/*N* * (%)	13/16 (81.3%)	8/15 (53.3%)	21/31 (67.7%)

* of *N* = 31 patients with an invasive procedure planned pre-Fibresolve and available data on management following Fibresolve testing.

## Data Availability

The original contributions presented in this study are included in the article. Restrictions apply to the availability of the raw datasets used—datasets were obtained from IMVARIA, Inc. and are available from the authors with the permission of IMVARIA, Inc.

## Abbreviations

The following abbreviations are used in this manuscript:

ILD	Interstitial lung disease
AI	Artificial intelligence
FDA	Food and Drug Administration
US	United States
IPF	Idiopathic pulmonary fibrosis
CTD-ILD	Connective tissue disease-related ILD
HP	Hypersensitivity pneumonitis
UIP	Usual interstitial pneumonia
FVC	Forced vital capacity
MDD	Multidisciplinary discussion
CT	Computed tomography
HRCT	High-resolution computed tomography
MDCT	Multi-detector computed tomography
CNN	Convolutional neural network
IQR	Interquartile range
CI	Confidence interval
GEE	Generalized estimating equation
OR	Odds ratio

## Appendix A

**Table A1:** Patient enrollment by geographic region.

Region	Academic Centers (Number of Patients)	Community-Based Centers (Number of Patients)	Total (Number of Patients)
Northeast United States	2 (9)	2 (25)	4 (34)
Midwest United States	3 (18)	4 (4)	7 (22)
Southern United States	4 (7)	14 (50)	18 (57)
Western United States	7 (23)	11 (73)	18 (96)
Total	16 (57)	31 (152)	47 (209)

**Table A2:** Baseline patient characteristics for all patients stratified by available data on pharmacotherapy pre- and post-Fibresolve utilization.

Characteristic	All Patients	Patients with Pre-Fibresolve Pharmacotherapy Data	Patients with Post-Fibresolve Pharmacotherapy Data
Age, median years (IQR) *	73 (67–79)	73 (67–78)	74 (68–78)
Sex, *n* (%) *			
Male	126 (60.3%)	100 (59.5%)	43 (59.7%)
Female	83 (39.7%)	68 (40.5%)	29 (40.3%)
Practice Setting, *n* (%) *			
Academic Center	57 (27.3%)	52 (30.9%)	25 (34.7%)
Community-Based Center	152 (72.7%)	116 (69.1%)	47 (65.3%)
Percent Predicted FVC, median (IQR) †	84% (71–95%)	84% (82–96%)	83% (70–94%)
Smoking Status, *n* (%) ‡			
Current Smoker	8 (4.6%)	4 (2.7%)	2 (3.3%)
Former Smoker	95 (54.3%)	81 (56.3%)	34 (55.7%)
Non-Smoker	72 (41.1%)	59 (41.0%)	25 (41.0%)

* *N* = 209 [168 with Pre-Fibresolve Data, 72 with Post-Fibresolve Data]; † *N* = 157 [138 with Pre-Fibresolve Data, 59 with Post-Fibresolve Data]; ‡ *N* = 175 [144 with Pre-Fibresolve Data, 61 with Post-Fibresolve Data]; IQR: interquartile range; FVC: forced vital capacity.

**Table A3:** Baseline patient characteristics for all patients stratified by available data on invasive diagnostic procedures pre- and post-Fibresolve utilization.

Characteristic	All Patients	Patients with Pre-Fibresolve Procedural Data	Patients with Post-Fibresolve Procedural Data
Age, median years (IQR) *	73 (67–79)	73 (67–78)	75 (68–78)
Sex, *n* (%) *			
Male	126 (60.3%)	94 (61.0%)	46 (59.7%)
Female	83 (39.7%)	60 (39.0%)	31 (40.3%)
Practice Setting, *n* (%) *			
Academic Center	57 (27.3%)	47 (30.5%)	26 (33.8%)
Community-Based Center	152 (72.7%)	107 (69.5%)	51 (66.2%)
Percent Predicted FVC, median (IQR) †	84% (71–95%)	84% (69–95%)	83% (70–94%)
Smoking Status, *n* (%) ‡			
Current Smoker	8 (4.6%)	4 (3.0%)	3 (4.8%)
Former Smoker	95 (54.3%)	68 (51.5%)	35 (55.6%)
Non-Smoker	72 (41.1%)	60 (45.5%)	25 (39.7%)

* *N* = 209 [168 with Pre-Fibresolve Data, 72 with Post-Fibresolve Data]; † *N* = 157 [126 with Pre-Fibresolve Data, 63 with Post-Fibresolve Data]; ‡ *N* = 175 [132 with Pre-Fibresolve Data, 63 with Post-Fibresolve Data]; IQR: interquartile range; FVC: forced vital capacity.

**Table A4:** Multivariable regression analysis assessing factors contributing to initiation of ILD-specific pharmacotherapy following Fibresolve testing.

Predictor	OR	95% CI	*p*-Value
Age (per year)	0.94	0.88–1.02	0.121
Sex	1.00	0.33–3.01	0.999
Ever-smoker	0.92	0.28–3.02	0.884
Percent predicted FVC (per unit)	0.98	0.95–1.01	0.232
Fibresolve result (positive vs. negative)	5.99	1.81–19.77	0.003

OR: odds ratio; CI: confidence interval; FVC: forced vital capacity

**Table A5:** Summary of the three FDA-approved artificial intelligence imaging platforms for use in interstitial lung disease.

Platform	Company	Output	Target Population
Fibresolve	IMVARIA	Identifies IPF using CT images; not limited to identification of UIP pattern	Patients undergoing workup of ILD (subtype not known)
IQ-UIP	4DMedical	Identifies a radiologic UIP pattern	Passive review of CT scans within a health system
e-Lung	Brainomix	Identifies and quantifies lung volume reduction/fibrosis	Patients with known ILD

IPF: idiopathic pulmonary fibrosis; CT: computed tomography; ILD: interstitial lung disease; UIP: usual interstitial pneumonia.
